# A Systematic Review on the Effect of Strontium-Doped Nanohydroxyapatite in Remineralizing Early Caries Lesion

**DOI:** 10.7759/cureus.44176

**Published:** 2023-08-26

**Authors:** Ratheesh Rajendran, Delphine P Antony, Princy Paul, Mohammed Ashik P, Ameena M, Hana Hameed

**Affiliations:** 1 Department of Conservative Dentistry and Endodontics, Saveetha College of Dental Sciences and Research, Chennai, IND; 2 Department of Conservative Dentistry and Endodontics, Saveetha Dental College, Saveetha Institute of Medical and Technical Sciences, Chennai, IND; 3 Department of Conservative Dentistry and Endodontics, Kunhitharuvai Memorial Charitable Trust (KMCT) Dental College, Kozhikode, IND; 4 Department of Oral Pathology and Microbiology, Azeezia College of Dental Sciences and Research, Kollam, IND; 5 Department of Conservative Dentistry and Endodontics, Krishnadevaraya College of Dental Sciences, Bengaluru, IND

**Keywords:** strontium-doped nanohydroxyapatite, srnhap dentifrices, dental decay, early caries lesions, tooth remineralization

## Abstract

The aim of this study is to review the potential of strontium-doped nanohydroxyapatite (SrnHAP) as a biomaterial for remineralizing early carious lesions. Publications from 2012 to 2022 were included based on the patient/population, intervention, comparison, and outcomes (PICO) framework, focusing on demineralized enamel treated with strontium-doped nanohydroxyapatite compared to other remineralizing agents, with the primary outcome being remineralization capacity. Electronic databases, namely, PubMed, Cochrane Library, and Google Scholar, were explored from March 31, 2023, to April 10, 2023. Only English language studies were included, while certain research types and studies on bovine teeth were excluded. Bias was assessed using the Cochrane methodology.

Five studies were synthesized, all using extracted human maxillary premolars. Four studies focused on remineralizing enamel, while one study focused on remineralizing dentin. Among these studies, comparisons were made between different strontium concentrations and various remineralizing agents such as nanohydroxyapatite (nHAP), Acclaim, casein phosphopeptide-amorphous calcium phosphate (CPP-ACP), and NovaMin. X-ray diffraction analysis was used to examine hydroxyapatite formation, while scanning electron microscopy (SEM) and transmission electron microscopy (TEM) were used for characterization. Additionally, one study evaluated the mechanical properties of partially demineralized dentin specimens. This study was registered in the PROSPERO under the ID CRD42023397413 and completed in accordance with the Preferred Reporting Items for Systematic Reviews and Meta-Analyses (PRISMA) guidelines.

## Introduction and background

Dental caries is a common dental disease affecting a major proportion of the worldwide population. Cavitation arises from the interaction between dietary fermentable carbohydrates and dental biofilm, leading to the demineralization of enamel and dentin. Appropriate preventive measures can remineralize early caries lesions [1,2]. Early or initial carious lesions are non-cavitated carious lesions limited to visual changes in enamel color and texture [3]. An uneven enamel surface and white spot lesions are common findings during orthodontic bonding and debonding. The progression in both cases can be prevented by the timely detection of early caries lesions and the application of appropriate remineralizing agents. Reversal of these lesions is the preventive measure for breaking further progression. Several investigators are in the experimental world in developing better remineralizing agents that can diffuse into the subsurface or deliver calcium and phosphorus into the subsurface. Restorative dentistry has adopted a more conservative approach in recent years, with remineralization procedures being the preferred method for regenerating lost tooth structure. Dental resources can be saved, and expenses and suffering for patients can be reduced when a preventive approach is taken to identify, preserve, and treat incipient caries [4].

New technologies for non-invasive treatment of caries have emerged, which focus on remineralizing early lesions. These include casein phosphopeptide-amorphous calcium phosphate (CPP-ACP), tricalcium phosphate (TCP), Pronamel, NovaMin, Enamelon, dicalcium phosphate dihydrate (DCPD), and ion-exchange resin (IER). CPP-ACP is a complex that deposits high concentrations of ACP close to the tooth surface, while TCP increases plaque fluid and saliva calcium and phosphate. Pronamel contains 5% potassium nitrate and 1,500 ppm NaF, making it both a desensitizing and remineralizing agent. NovaMin is a bioactive glass that provides calcium and phosphate through hydroxyl carbonate apatite formation. Enamelon treats white spot lesions and repairs and remineralizes tooth enamel. DCPD is an abrasive that improves the effects of fluoride, while IER is a controlled-release system for anticaries treatment [5].

Various fluoride devices have been developed to prevent dental caries. The first is a slowly dissolving fluoride glass device that releases fluoride when moist in saliva and is adhered to the surface of the first permanent molar using adhesive resins. A new disk-shaped device has been introduced, which is placed inside a plastic bracket. Another device is a copolymer membrane-controlled reservoir type that releases fluoride in the range varying from 0.02 to 1 mgF/day for up to 180 days. To overcome the slow release of fluoride, a dental bracket and associated kit have been introduced that attaches a pellet to the tooth that can release the fluoride [5]. A new dicalcium phosphate anhydrous (DCPA) nanocomposite has also been developed that slowly releases high levels of calcium phosphate for remineralization purposes [6]. A recent introduction is the use of specialized phosphonate-containing polymers or telomeres that enhance fluoride incorporation into the tooth. Other devices include a hydroxyapatite-Eudragit RS 100 diffusion-controlled fluoride system and topical fluorides such as amine fluoride and stannous hexafluorozirconate. Laser treatment has also been found to have a synergistic effect with fluoride.

Hydroxyapatite nanoparticles (nHAP) have the same structure and appearance as the enamel crystals, enabling them to simulate the natural mineral content of enamel and repair it [7]. Research indicates that particles of nHAP measuring 20 nm are effective in plugging the minute pores on the enamel surface formed due to acid erosion. These nanoparticles strongly adhere to the demineralized area and prevent further acid destruction [4]. Toothpaste infused with nHAP considerably reduces tooth sensitivity by blocking dentin tubules. It is more beneficial than traditional fluoride in repairing initial enamel lesions since it has free calcium, essential for remineralization and defense against dental erosion and caries [8]. Studies show that the use of strontium-doped nanohydroxyapatite (SrnHAP) ceramics in ultraviolet light-emitting diode (UV-LED) cured dental composites resulted in a more profound curing depth due to their high radiopacity. The substitution of strontium is expected to enhance the antibacterial characteristics and ability to promote remineralization [9-11].

Strontium is an alkaline earth metal that occurs naturally and has an extensive range of actions from treating and preventing osteoporosis to managing dentinal hypersensitivity in the oral cavity. It exhibits properties such as cariostatic, antiplaque, and antigingivitis [12]. The addition of strontium to calcium-containing HA leads to the formation of pure, non-stoichiometric HA with low (Sr+Ca)/P ratios. According to earlier research, 25% strontium-doped nanohydroxyapatite (SrnHAP) had a particle size ranging from 2 to 7 µm, whereas 50% SrnHAP has a particle size of 4-9 µm. Due to its larger particle size, 50% SrnHAP is not a suitable candidate for creating a paste system to be applied over the enamel surface. Hence, 25% SrnHAP is the preferred material for use in tiny incipient caries lesions [13].

There is currently no systematic review that has evaluated the efficacy of this agent in clinical trials. Therefore, a systematic review is needed to provide an objective assessment of the available evidence and determine whether SrNHA is an effective agent for remineralizing early carious lesions. This also helps to identify the strengths and weaknesses of the available studies on SrNHA. This can help to inform clinical decision-making and guide future research in this area and can contribute to the development of more effective preventive and therapeutic interventions for dental caries.

## Review

Methods

Search Strategy

The study was registered in the PROSPERO under the ID CRD42023397413 and completed in accordance with Preferred Reporting Items for Systematic Reviews and Meta-Analyses (PRISMA) guidelines [14]. Three electronic databases were searched: PubMed, Cochrane Library, and Google Scholar. The eligibility criteria included in vitro studies conducted on the application of strontium nanohydroxyapatite for remineralization of early carious lesions in the enamel. The first stage of the study selection was carried out by reading the titles and abstracts. The remaining articles were read in full to verify eligibility. From this stage, the papers selected for this review were defined, and a manual search was carried out in the references to find other eligible articles. Two independent authors performed the search and selection of studies, and a third reviewer evaluated the data in cases of disagreement.

Selection Criteria

A systematic exploration of the literature was conducted using pertinent keywords to answer the research question at hand. The three main concepts that were of interest were SrnHAP, remineralization, and early caries lesions. Various search terms, such as MeSH terms, keywords, and related terms, were used to conduct the literature search, focusing on studies involving SrnHAP and dentifrices (Table 1). Other potentially relevant publications were manually reviewed, and the references of relevant studies were scrutinized. The search covered publications from January 2012 to December 2022, and the titles and abstracts of these publications were scanned for pertinent keywords.

**Table 1:** Search strategy in PubMed MeSH: Medical Subject Headings

Query	Results
(dental caries[MeSH Terms]) OR ((Tooth remineralization [MeSH Terms]) OR (remineralization, tooth [MeSH Terms]))	51,174
(Strontium [MeSH Terms]) AND (((“nano” [Journal] OR “nano” [All Fields]) AND (“durapatite” [MeSH Terms] OR “durapatite” [All Fields] OR “hydroxyapatite” [All Fields] OR “hydroxyapatites” [MeSH Terms] OR “hydroxyapatites” [All Fields])) AND (2012:2023 [pdat]))	30
((“dentifrices” [Pharmacological Action] OR “dentifrices” [MeSH Terms] OR “dentifrices” [All Fields] OR “dentifrice” [All Fields]) AND (2012:2023 [pdat])) AND ((“dentifrices” [Pharmacological Action] OR “dentifrices” [MeSH Terms] OR “dentifrices” [All Fields] OR “dentifrice” [All Fields]) AND (2012:2022 [pdat]))	2,457

The keywords used in Google Scholar and Cochrane Library were “strontium-doped nanohydroxyapatite,” “tooth remineralization,” and “early caries lesions.”

Data Abstraction

The initial stage of the study selection process encompassed scrutinizing the titles and abstracts of articles to recognize potentially relevant ones. To guarantee that the studies fulfilled the inclusion criteria, the remaining articles underwent scrupulous examination. Ultimately, the studies that met the eligibility criteria were distinguished, and an additional search was carried out to identify any relevant studies that might have been missed earlier. This entire search and selection process was conducted independently by two reviewers, and any discrepancies were solved by a third reviewer.

An exhaustive evaluation of the studies was conducted to obtain pertinent information such as authors, country of publication, study design, tooth type, assessment and management of demineralization and remineralization, and results, which were documented using an Excel sheet. Whenever the data was missing, the authors were contacted by email every week for up to a month to obtain the information. The selected studies were analyzed using Review Manager software [15].

Results

Study Characteristics

**Figure 1:**
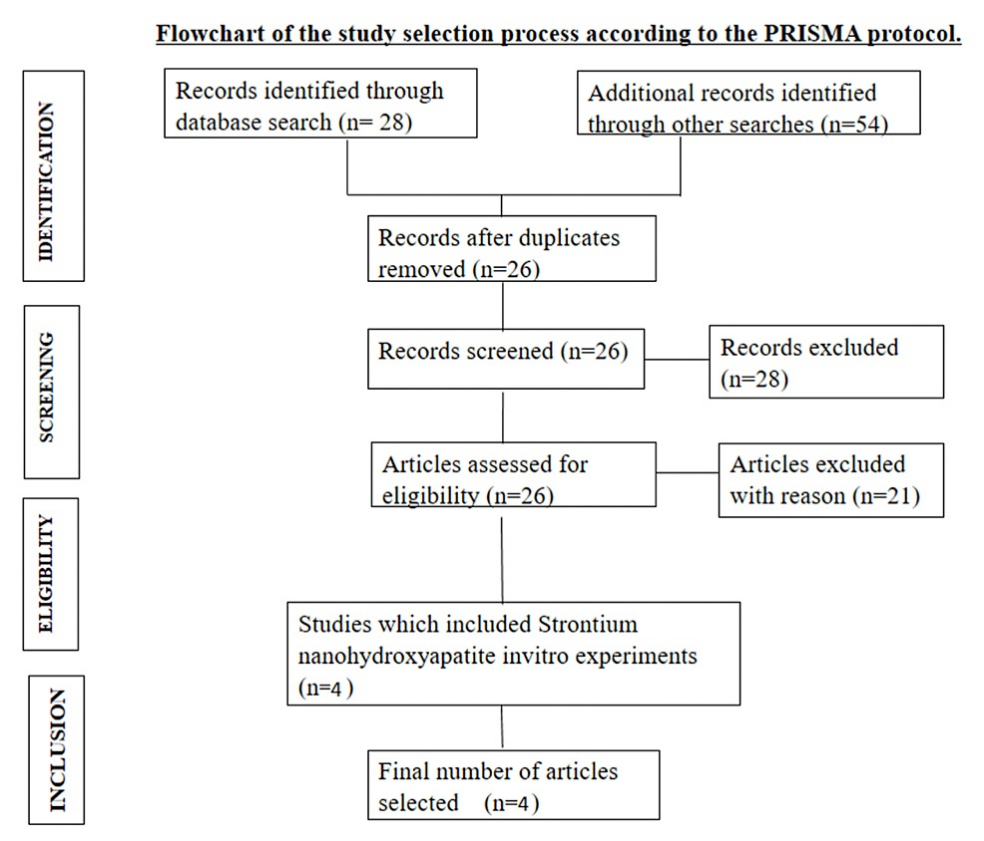
Flowchart of the study selection process according to the PRISMA protocol PRISMA: Preferred Reporting Items for Systematic Reviews and Meta-Analyses

Out of the 82 studies that satisfied the criteria for inclusion, merely four were selected for the synthesis (Figure 1). Only in vitro experiments published during the period from 2012 to 2022 were included in the study. Four studies included in the synthesis (Table 2) used extracted human maxillary premolars [13,16-18]. Four studies focused on remineralizing enamel [13,16-18]. Among the studies, one compared 10% and 20% strontium concentrations, and another compared 25% and 50% concentrations, along with nHAP, Acclaim, and ACP-CPP that was custom prepared. In the remaining three studies, SrnHAP was compared to other existing remineralizing agents such as NovaMin and casein phosphopeptide-amorphous calcium phosphate. X-ray diffraction analysis was the most commonly used method to elicit hydroxyapatite formation, while scanning electron microscopy (SEM) and transmission electron microscopy (TEM) were used to characterize the results.

**Table 2:** Studies included Sr: strontium, nHAP: nanohydroxyapatite, CPP-ACP: casein phosphopeptide-amorphous calcium phosphate, ACP-CPP: amorphous calcium phosphate-casein phosphopeptide, ANOVA: analysis of variance, SEM: scanning electron microscopy, EDX: dispersive X-ray

Author	Size and source of sample	Teeth	Remineralization evaluation	Study groups	Results
Krishnan et al. (2016) [13]	50	Intact premolars	Atomic force microscopy, SEM, and microindentation testing	Group I: control/no treatment, group II: ACP-CPP-based solution used, Group III: Acclaim (commercially available nHAP, Group Pharmaceutical Limited), group IV: nHAP (custom prepared), group V: 25 mol% Sr-doped nHAP group VI: 50 mol% Sr-doped nHAP	The mean and standard deviation values of the Vickers micro-hardness from the dental specimens of all six groups and the results of one-way ANOVA show that the specimens treated with 50% strontium-doped nHAP possess greater hardness in comparison with all other specimens (p=0.001). The increase in crystallinity and reduced particle size favored dissolution and re-precipitation through small incipient carious lesions and soft white spot areas with 25% Sr-doped nHAP. Sr-doped specimens showed more cell viability in comparison with pure nHAP.
Rajendran et al. (2020) [16]	60	Intact premolars	Cytotoxicity by direct microscopic observation and MTT assay remineralization by SEM and EDX analysis	Group I: regular toothpaste, group II: SrnHAP paste	Statistical analysis was done using one-way ANOVA and Tukey’s post hoc test. Intergroup comparison of group I and group II after remineralization was done using Student’s t-test (p<0.001). The result of the study concluded that SrnHAP paste showed better remineralization potential and favorable surface changes in enamel compared to the regular dentifrice.
Rajendran et al. (2021) [17]	90	Intact premolars	SEM and EDX analysis	Group I: conventional toothpaste, group II: CPP-AMP-containing toothpaste, group III: SrnHAP paste	The novel laboratory-synthesized Sr‑doped nanohydroxyapatite showed better remineralization than CPP‑ACP (one-way ANOVA and Tukey’s post hoc test: p<0.001).
Rajendran et al. (2022) [18]	120	Intact premolars	SEM and EDX analysis	Group I: conventional toothpaste (control group), group II: calcium sodium phosphosilicate (NovaMin), group III: CPP-ACP (GC tooth mousse), group IV: novel strontium-doped nanohydroxyapatite paste (SrnHAP paste)	Kruskal‑Wallis, ANOVA, and Mann-Whitney tests showed significant p values. The novel laboratory-synthesized SrnHAP showed better remineralization compared to other groups with GC tooth mousse and NovaMin.

Outcomes

The tooth samples used underwent a process of demineralization, followed by the application of different remineralizing agents, including the laboratory-synthesized SrnHAP. In the study conducted by Krishnan et al. in 2016, 50 tooth samples were divided into six groups and treated with various remineralizing agents, including ACP-CPP, commercially available nHAP, and 25% and 50% SrnHAP, and a control group that received no remineralizing agents. The results showed that 25% SrnHAP had the best remineralization capacity compared to the other groups [13].

Rajendran et al. also conducted various research about the remineralizing properties of SrnHAP. In one study, the researchers evaluated SrnHAP using SEM and EDX analysis and compared it with regular toothpaste. The outcome of the study showed that SrnHAP presented better remineralization compared to conventional toothpaste. In another study, SrnHAP was compared with CPP-ACP and conventional toothpaste, and the results showed that SrnHAP had better remineralization [16,17].

Finally, SrnHAP was assessed with two remineralizing agents by Rajendran et al. in 2022, and the results highlighted the potential of SrnHAP as a better alternative to other remineralizing agents. Overall, the studies demonstrate the potential of SrnHAP as an effective remineralizing agent for the treatment of demineralized teeth [18].

Discussion

Caries that spread to the junction between the enamel and dentin, as well as non-cavitated lesions, can be halted by regulating the cariogenic needs of the specific microenvironment appropriately or by administering drugs to promote tissue regeneration. Various remineralizing agents have been employed to increase the likelihood of remineralization of demineralized enamel. In one study, a unique form of SrnHAP crystals was created and compared to a remineralizing agent, CPP-ACP. Samples were demineralized using McInne’s demineralizing solution, then treated with specific remineralizing agents, and kept in artificial saliva for 28 days to complete the remineralization process. After remineralization and demineralization, the mean calcium and phosphorus contents of the specimens were measured using SEM and EDX and compared to CPP-ACP for remineralization and repair of enamel demineralization. The presence of strontium indicated higher solubility and better adhesion to the tooth surface [17].

Similar results were found in another study that showed that CPP-ACP cream and nHAP-containing toothpaste were not as effective as SrnHAP for restoring demineralized enamel surfaces. The study found that Sr-doped specimens have increased crystallinity and reduced particle size, which is beneficial for dissolving and re-precipitating through small, early-stage caries lesions and soft white spot areas. Moreover, these specimens demonstrated greater cell viability than pure nHAP, indicating that they are biologically compatible for use in patients’ mouths [13].

Bioactive glass is compared with SrnHAP and CPP-ACP to evaluate the efficacy of enamel remineralization. All groups exhibited a positive gain in calcium and phosphorous content after the administration of their respective remineralizing agents. Comparing the mean calcium/phosphorus ratios of each group, it was determined that SrnHAP paste produced a greater increase in net calcium and phosphorous values than the other groups [18].

The replacement of calcium with strontium in bioactive materials has shown potential benefits. However, there have been certain challenges related to the instability of the product and the possibility of its toxicity [19]. Cytotoxic evaluation of the SrnHAP dentifrice was performed in a study. MTT assay and lethal concentration 50% (LC50) values proved that the novel paste had improved cell viability and reduced cytotoxicity than the daily dentifrice [16].

To evaluate the effect of the gradient in Sr ion concentration on enamel demineralization, studies were carried out. The enamel was acidified after the release of calcium ions and phosphate ions; therefore, the surface of the enamel was negatively charged. This negatively charged enamel surface could attract a great amount of positively charged strontium ions onto the enamel surface; this layer of positively charged strontium ions was called the Stern layer [20]. The findings in studies suggested that Sr ions may create a Stern layer, which reduces the acidic solution’s inclination for corrosion and bestows a defensive role on the enamel [20]. The author also cited a study that showed that toothpaste with strontium could elevate the amount of strontium in enamel and decrease its susceptibility to dissolving [14]. According to the study, toothpaste containing Sr, F-, and Sr-HAP used for six months could remineralize the tooth’s white spot lesion [21,22].

From the literature, the nHAP doped with Sr demonstrated superior remineralization than many other commercially available remineralizing agents [22].

## Conclusions

A comprehensive understanding of demineralization and remineralization stages is necessary to develop a competent remineralizing agent. However, further in vivo studies and clinical trials are required to prove the suitability of SrnHAP in clinical scenarios. It is very essential to evaluate the long-term merits of its application in the oral cavity. Research is also needed to optimize the incorporation of Sr into nHAP to improve its antibacterial properties and promote remineralization. This may lead to the development of innovative dental products that are more effective in repairing and protecting teeth against erosion and caries. Combining Sr-doped nHAP with other remineralizing agents such as F- could also enhance its effectiveness in repairing enamel lesions. Overall, the development of Sr-doped nHAP in dentistry holds great promise for improving oral health and preventing dental disease.
